# Supplementary material for: Role and mechanism of NCAPD3 in promoting malignant behaviors in gastric cancer
Source: Front Pharmacol. 2024 Apr 22;15:1341039. doi: 10.3389/fphar.2024.1341039 (PMC11070777; doi:10.3389/fphar.2024.1341039)
Supplement: Supplementary file 11 [file DataSheet2.ZIP › GSEA/Transcipt factor/my_analysis.Gsea.1599461814734/ZSCAN31_TARGET_GENES.html]

Details for gene set ZSCAN31\_TARGET\_GENES[GSEA]

|  || Dataset | filtered\_dataset.sample\_info.cls#WT\_versus\_NCAPD3\_MUT |
| Phenotype | sample\_info.cls#WT\_versus\_NCAPD3\_MUT |
| Upregulated in class | WT |
| GeneSet | ZSCAN31\_TARGET\_GENES |
| Enrichment Score (ES) | 0.2504322 |
| Normalized Enrichment Score (NES) | 1.6506495 |
| Nominal p-value | 0.007824726 |
| FDR q-value | 0.5150424 |
| FWER p-Value | 1.0 |
Table: GSEA Results Summary

  

Fig 1: Enrichment plot: ZSCAN31\_TARGET\_GENES      
 Profile of the Running ES Score & Positions of GeneSet Members on the Rank Ordered List

  

| SYMBOL | TITLE | RANK IN GENE LIST | RANK METRIC SCORE | RUNNING ES | CORE ENRICHMENT || 1 | 222236 | NAPEPLD | 36 | 0.977 | -0.0064 | Yes |
| 2 | 57594 | HOMEZ | 44 | 0.933 | 0.0082 | Yes |
| 3 | 388536 | ZNF790 | 52 | 0.913 | 0.0223 | Yes |
| 4 | 57542 | KLHL42 | 59 | 0.876 | 0.0364 | Yes |
| 5 | 2120 | ETV6 | 63 | 0.862 | 0.0524 | Yes |
| 6 | 642852 | LOC642852 | 64 | 0.860 | 0.0707 | Yes |
| 7 | 157680 | VPS13B | 78 | 0.838 | 0.0787 | Yes |
| 8 | 284323 | ZNF780A | 90 | 0.805 | 0.0875 | Yes |
| 9 | 81839 | VANGL1 | 98 | 0.790 | 0.0990 | Yes |
| 10 | 55006 | TRMT61B | 101 | 0.782 | 0.1141 | Yes |
| 11 | 55687 | TRMU | 128 | 0.774 | 0.1109 | Yes |
| 12 | 26272 | FBXO4 | 133 | 0.767 | 0.1242 | Yes |
| 13 | 390927 | ZNF793 | 137 | 0.758 | 0.1381 | Yes |
| 14 | 55900 | ZNF302 | 145 | 0.740 | 0.1485 | Yes |
| 15 | 7733 | ZNF180 | 154 | 0.728 | 0.1579 | Yes |
| 16 | 2186 | BPTF | 159 | 0.717 | 0.1702 | Yes |
| 17 | 134957 | STXBP5 | 170 | 0.709 | 0.1777 | Yes |
| 18 | 54971 | BANP | 172 | 0.707 | 0.1919 | Yes |
| 19 | 84911 | ZNF382 | 182 | 0.692 | 0.1999 | Yes |
| 20 | 51018 | RRP15 | 192 | 0.685 | 0.2076 | Yes |
| 21 | 3308 | HSPA4 | 194 | 0.685 | 0.2214 | Yes |
| 22 | 84190 | METTL25 | 195 | 0.684 | 0.2360 | Yes |
| 23 | 6191 | RPS4X | 196 | 0.682 | 0.2504 | Yes |
| 24 | 403313 | PLPP6 | 274 | 0.615 | 0.2055 | No |
| 25 | 129831 | RBM45 | 310 | 0.587 | 0.1916 | No |
| 26 | 147929 | ZNF565 | 311 | 0.587 | 0.2040 | No |
| 27 | 51491 | NOP16 | 334 | 0.573 | 0.1996 | No |
| 28 | 57109 | REXO4 | 370 | 0.552 | 0.1850 | No |
| 29 | 115509 | ZNF689 | 374 | 0.551 | 0.1944 | No |
| 30 | 10426 | TUBGCP3 | 402 | 0.535 | 0.1854 | No |
| 31 | 801 | CALM1 | 424 | 0.518 | 0.1806 | No |
| 32 | 55870 | ASH1L | 436 | 0.512 | 0.1832 | No |
| 33 | 57478 | USP31 | 448 | 0.506 | 0.1857 | No |
| 34 | 25880 | TMEM186 | 462 | 0.498 | 0.1865 | No |
| 35 | 5245 | PHB | 481 | 0.486 | 0.1832 | No |
| 36 | 23468 | CBX5 | 484 | 0.485 | 0.1920 | No |
| 37 | 1819 | DRG2 | 550 | 0.447 | 0.1525 | No |
| 38 | 79707 | NOL9 | 560 | 0.441 | 0.1551 | No |
| 39 | 6632 | SNRPD1 | 563 | 0.440 | 0.1630 | No |
| 40 | 84775 | ZNF607 | 564 | 0.440 | 0.1723 | No |
| 41 | 4548 | MTR | 592 | 0.428 | 0.1611 | No |
| 42 | 23195 | MDN1 | 596 | 0.426 | 0.1679 | No |
| 43 | 7290 | HIRA | 612 | 0.417 | 0.1654 | No |
| 44 | 10445 | MCRS1 | 617 | 0.414 | 0.1712 | No |
| 45 | 5147 | PDE6D | 624 | 0.410 | 0.1754 | No |
| 46 | 643836 | ZFP62 | 639 | 0.405 | 0.1734 | No |
| 47 | 79807 | GSTCD | 644 | 0.402 | 0.1790 | No |
| 48 | 51728 | POLR3K | 646 | 0.401 | 0.1867 | No |
| 49 | 78996 | C7orf49 | 661 | 0.396 | 0.1846 | No |
| 50 | 6427 | SRSF2 | 679 | 0.388 | 0.1800 | No |
| 51 | 57466 | SCAF4 | 724 | 0.361 | 0.1545 | No |
| 52 | 153562 | MARVELD2 | 732 | 0.359 | 0.1568 | No |
| 53 | 128061 | C1orf131 | 738 | 0.356 | 0.1606 | No |
| 54 | 84451 | KIAA1804 | 751 | 0.348 | 0.1590 | No |
| 55 | 27300 | ZNF544 | 759 | 0.343 | 0.1610 | No |
| 56 | 54939 | COMMD4 | 777 | 0.335 | 0.1553 | No |
| 57 | 29960 | FTSJ2 | 805 | 0.319 | 0.1417 | No |
| 58 | 5898 | RALA | 813 | 0.309 | 0.1430 | No |
| 59 | 4285 | MIPEP | 815 | 0.307 | 0.1488 | No |
| 60 | 3146 | HMGB1 | 831 | 0.295 | 0.1437 | No |
| 61 | 9524 | TECR | 834 | 0.292 | 0.1484 | No |
| 62 | 8835 | SOCS2 | 840 | 0.275 | 0.1505 | No |
| 63 | 54206 | ERRFI1 | 849 | -0.229 | 0.1494 | No |
| 64 | 7357 | UGCG | 852 | -0.254 | 0.1532 | No |
| 65 | 3673 | ITGA2 | 858 | -0.264 | 0.1551 | No |
| 66 | 51278 | IER5 | 874 | -0.283 | 0.1498 | No |
| 67 | 9141 | PDCD5 | 958 | -0.379 | 0.0953 | No |
| 68 | 3669 | ISG20 | 1097 | -0.475 | 0.0014 | No |
| 69 | 9236 | CCPG1 | 1128 | -0.500 | -0.0106 | No |
| 70 | 831 | CAST | 1160 | -0.524 | -0.0228 | No |
| 71 | 6430 | SRSF5 | 1183 | -0.553 | -0.0277 | No |
| 72 | 9113 | LATS1 | 1201 | -0.577 | -0.0282 | No |
| 73 | 9709 | HERPUD1 | 1202 | -0.578 | -0.0159 | No |
| 74 | 219790 | RTKN2 | 1204 | -0.579 | -0.0044 | No |
| 75 | 4651 | MYO10 | 1213 | -0.588 | 0.0021 | No |
| 76 | 440 | ASNS | 1232 | -0.609 | 0.0014 | No |
| 77 | 135114 | HINT3 | 1257 | -0.645 | -0.0029 | No |
| 78 | 8916 | HERC3 | 1269 | -0.660 | 0.0028 | No |
| 79 | 7994 | KAT6A | 1279 | -0.666 | 0.0102 | No |
| 80 | 25938 | HEATR5A | 1316 | -0.730 | -0.0015 | No |
| 81 | 57561 | ARRDC3 | 1324 | -0.744 | 0.0091 | No |
| 82 | 10611 | PDLIM5 | 1335 | -0.758 | 0.0176 | No |
| 83 | 255488 | RNF144B | 1362 | -0.834 | 0.0158 | No |
| 84 | 6711 | SPTBN1 | 1381 | -0.925 | 0.0219 | No |
Table: GSEA details [plain text format]

  

Fig 2: ZSCAN31\_TARGET\_GENES      
 Blue-Pink O' Gram in the Space of the Analyzed GeneSet

  

Fig 3: ZSCAN31\_TARGET\_GENES: Random ES distribution      
 Gene set null distribution of ES for **ZSCAN31\_TARGET\_GENES**
